# University Students’ Lifestyle Behaviors during the COVID-19 Pandemic: A Four-Wave Longitudinal Survey

**DOI:** 10.3390/ijerph18178998

**Published:** 2021-08-26

**Authors:** Aurélie Goncalves, Sarah Le Vigouroux, Elodie Charbonnier

**Affiliations:** UNIV. NIMES, APSY-V, F-30021 CEDEX Nîmes, France; sarah.le_vigouroux_nicolas@unimes.fr (S.L.V.); elodie.charbonnier@unimes.fr (E.C.)

**Keywords:** coronavirus, sedentary behavior, physical activity, alcohol, university, lockdown, longitudinal survey

## Abstract

Many studies have highlighted the impact of lockdowns on the lifestyle of university students. We do not, however, know how these lifestyles have changed over the course of the COVID-19 pandemic. The objective of the present study was to describe changes in the levels of physical activity, sedentary behaviors, and alcohol consumption in French university students during different periods of the pandemic. This cohort study was conducted between 23 April and 11 December 2020. Measurements were performed four times: Twice during two successive lockdowns and twice during the intervening period. A total of 1294 university students were initially included, and 91 students completed the four measurement points over a seven-month period. Alcohol consumption, physical activity, and sedentary behaviors were measured. The results revealed high levels of physical activity during the first lockdown, but a subsequent decline during the pandemic that was partly explained by time. The pandemic had a positive effect on alcohol consumption. Sedentary levels were higher during both lockdowns, and sedentary behaviors tended to persist over time. This study raises concerns about the long-term effects of the pandemic on students’ health and lifestyle. The preponderance of distance learning should be reconsidered, given the negative impact of physical inactivity and sedentary behavior on long-term health, especially in young adults.

## 1. Introduction

The COVID-19 pandemic has had huge repercussions around the world. The measures taken to reduce the circulation of the virus, such as reducing travel, closing sports halls, imposing lockdowns, and encouraging employees to work from home, have led to profound and rapid changes in people’s lifestyles. More specifically, during the first lockdown, there was an increase in sedentary behaviors, a reduction in physical activity, and a stronger tendency to eat an unhealthy diet [1,2,3]. At the same time, on 25 November 2020, the World Health Organization [4] published new recommendations on physical activity and sedentary behaviors, and reaffirmed their key role in protecting and promoting health. Among the recommendations for adults 18–64 years of age, they should (for substantial health benefits): (1) Undertake regular physical activity; (2) do at least 150–300 min of moderate-intensity aerobic physical activity or at least 75–150 min of vigorous intensity aerobic physical activity, or an equivalent combination of moderate- and vigorous-intensity activity throughout the week; (3) limit the amount of time spent being sedentary, replacing sedentary time with physical activity of any intensity (including light intensity). Beyond its direct impact on health, the pandemic may therefore also have had an indirect impact on health by modifying people’s lifestyles.

Even before the pandemic, students were identified as a population with unhealthy lifestyles and habits [5], notably reflected in high levels of sedentary behaviors, low levels of physical activity [6,7], and an unhealthy diet [8]. University students have escaped neither COVID-19 infections nor other repercussions of the pandemic [9,10,11]. In France, all universities closed on 16 March 2020 and the country was strictly locked down with 1 h of authorized exit per day from 17 March to 10 May 2020. In September 2020, face-to-face teaching resumed in French universities, but with new constraints (e.g., fewer students in classrooms and mask wearing) and major changes in teaching (e.g., distance and/or hybrid education). In October 2020, several French universities closed again, owing to the high infection rates among students, and on 30 October 2020, the French Government imposed a second national lockdown and all universities had to close again. The lockdown ended on 15 December 2020, but universities remained closed to students, except for a few courses involving practical work. Face-to-face teaching was partially (approximately 20%) resumed in February 2021. University students have encountered many educational challenges (e.g., widespread transition to remote online learning, changes in assessment and examinations, and disruption to internship placements), which have had a major impact on their habits. During the first lockdown, some studies conducted on the general population [1,12,13] and others studies conducted among students reported a decrease in alcohol consumption, expressed as a reduction in binge drinking [14,15], as well as in the frequency of drinking [16,17]. For example, 68.2% of Belgian students stated that they had consumed less alcohol during the first lockdown, compared to previous levels [16]. Similarly, a longitudinal study [17] conducted in the United States over three springs (2018, 2019, and 2020) showed that college students did not increase their drinking frequency in 2020 as was typical at the end of the spring semester (in 2018 and 2019). In addition, this study also highlighted that their number of drinks per occasion decreased significantly (28% reduction) during the onset of the COVID-19 pandemic. Furthermore, in France, a substantial decrease in binge drinking was observed during the first lockdown (prevalence decreased from 35.9% to 9.3%) [15]. However, it is important to note that, to a lesser extent, some studies [18,19] conducted among students reported contradictory data. Indeed, in the United States, a retrospective study was highlighted that alcohol consumption increased after the closure of the campus [18]. In the same vein, the authors of [19] highlighted an increase in drinking frequency during lockdown; however, this study also showed a decrease in quantity, binge drinking and drunkenness. In other words, although there is no consensus, the majority of the literature tends to point to a decrease in alcohol consumption during lockdown. Major changes in students’ physical activity have also been observed during the lockdowns, but with very heterogeneous results (some studies reporting an increase in physical activity, others a decrease) and high variability according to participant profiles (e.g., sex and physical activity level before lockdown) [20,21,22,23,24]. Across nationalities, the results are also contradictory, with, for example, a reported increase in weekly physical activity among Spanish students [25], but a 30% decrease among Australian students [21] and a 29.5% decrease in moderate physical activity among Italian students [22]. In France, during the first lockdown, a decrease in physical activity was highlighted for a third of the students, and an increase for a quarter of them [15]. Finally, it has been reported that the lockdowns have increased sedentary behaviors, especially among students, who have seen their opportunities for travel disappear almost completely and their screen time increase substantially, with a 2 h increase in daily sitting time among Spanish students [25] and an increase in sedentary time of up to 50% among Italian students [22]. Along the same line, it was found that sitting and sedentary behaviors significantly increased among university students in Bangladesh during lockdown [26]. COVID-19 has brought an increase in the average number of hours (8.3 to 11 h) students spend engaging in sedentary activities [27].

In summation, the literature findings show that the beginning of the pandemic—more specifically, the first lockdown—clearly had a massive impact on the lifestyle of university students, as evidenced by their high levels of sedentary behaviors and changes in alcohol consumption and physical activity. However, we do not yet know what impact the months following the initial lockdown have had, and what challenges will be faced by students in the months and years to come. The objective of the present study was to describe changes in the levels of physical activity, sedentary behaviors, and alcohol consumption in French university students during different periods of the COVID-19 pandemic (two lockdowns and the intervening period). Following the closure of bars and the limitation of festive events during the lockdowns, and in accordance with the results of previous study that were conducted during the first lockdown [16], we hypothesized that during the lockdowns, compared to the intervening period, university students drank less alcohol (H1). In addition, in line the findings from the first lockdown [21,22,28], we also hypothesized that students engaged in less physical activity (H2) and more sedentary behaviors (H3) during the lockdowns.

## 2. Materials and Methods

### 2.1. Study Design

Data were collected anonymously at four timepoints between 23 April and 11 December 2020 via an online survey designed with Qualtrics software (Qualtrics, Provo, UT, USA). A link to the survey was sent by e-mail to teachers in different faculties at a number of French universities. The link to the survey was also distributed via students’ social media (e.g., Facebook groups). Our only criterion for inclusion was to be a student at a French university. Participants agreed to participate in this study after reading the consent form. They were informed that their personal data would remain anonymous and that their participation was voluntary, and they could withdraw at any time. All of the procedures contributing to this work were undertaken in compliance with the ethical standards of the relevant national and institutional committees on human experimentation and with the 1975 Declaration of Helsinki, revised in 2008. No compensation was offered to participants.

To track changes in students’ lifestyle, we carried out measures at four timepoints: (1) During France’s first national lockdown, between 23 April and 8 May 2020 (N_T1_ = 1294; M_age_ = 21.28 years, SD = 4.73); (2) after the first lockdown, between 9 and 23 June 2020 (N_T2_ = 373; M_age_ = 22.12 years, SD = 5.70); when the virus was under control and the universities were open, between 12 and 23 October 2020 (N_T3_ = 284; M_age_ = 21.95 years, SD = 5.33); and during the second lockdown, between 20 November and 11 December 2020 (N_T4_ = 160; M_age_ = 21.81 years, SD = 6.05). At the end of the first questionnaire, participants were asked to complete a code that guaranteed their anonymity and allowed us to cross-match the data between waves. A total of 91 participants, aged between 18 and 51 years old (M_age_ = 22.35, SD = 5.84), responded at all time points. Their characteristics are set out in Table 1. In addition, for information only, the characteristics of all participants (i.e., who responded to only one, two, or three times) are presented in Table S1.

### 2.2. Measures

Alcohol consumption was assessed using the Alcohol Use Disorders Identification Test-Consumption (AUDIT-C [29]) scale. Participants rated its three items on a 5-point scale. These items probed the frequency of alcohol consumption, with scores ranging from 0 (Never) to 4 (At least 4 times a week); the amount of alcohol consumed, with scores ranging from 0 (1 or 2) to 4 (10 or more); and the frequency of binge drinking (i.e., more than 6 drinks during a single occasion), with scores ranging from 0 (Never) to 4 (Almost every day) over the previous month. Higher scores reflect higher levels of alcohol use. During the first assessment, participants answered the AUDIT-C for both the previous month (i.e., during lockdown) and their consumption before the lockdown (i.e., retrospectively).

Physical activity and sedentary behaviors were assessed using a modified version of the Recent Physical Activity Questionnaire (RPAQ) that had previously been used in a French epidemiological study [30]. The modified RPAQ assesses physical activity over the previous 4 weeks in the following four domains: At home, during travel, at work, and during leisure and sport. Activity is classified as follows: Sedentary time, light physical activity (walking slowly or household activities; e.g., sweeping the floor, vacuuming, and washing windows), moderate physical activity (active travel or moderately intense leisure activities; e.g., walking briskly and strength training at home), and vigorous physical activity (e.g., running and swimming).

### 2.3. Statistical Analyses

In order to test the differences between our four times (before pandemic, T1, T2, T3, and T4) on physical activity, sedentary behaviors, and alcohol consumption, repeated measures ANOVAs were used.

## 3. Results

**Figure 1:**
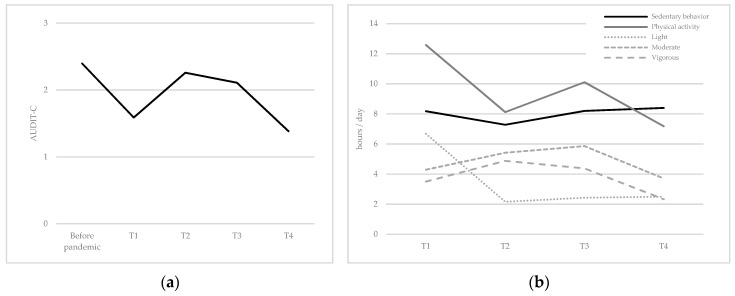
Mean (**a**) alcohol consumption and (**b**) physical activity.

The results (Table 2 and Table 3 and Figure 1a) showed changes over time in alcohol consumption. In line with H1, we observed a decrease in alcohol consumption during each lockdown (T1 and T4). In contrast, the consumption levels were similar before the pandemic, and during the deconfinement periods (T2 and T3). Moreover, our results on physical activity (Table 2 and Table 3 and Figure 1b) showed changes over time in total physical activity. More specifically, we observed extremely high levels of physical activity during the first lockdown (T1), but these decreased significantly as soon as the lockdown was lifted (T2). There was a slight increase when classes at university resumed (T3), but a further decrease during the second lockdown (T4). In other words, H2 was partially validated, as physical activity levels were indeed lower during the second lockdown, but not during the first. Interestingly, our analyses showed differences in light physical activity but not in moderate (except between T3 and T4) or vigorous physical activity. Accordingly, high levels of light physical activity during the first lockdown (T1) fell drastically thereafter (T2–T4). Finally, our results highlighted few differences in sedentary behavior. Consistent with H3, sedentary levels were slightly lower after the first lockdown and the end of classes (T2) compared to all other times. Contrary to our expectations, the level of sedentary behaviors increased when classes resumed (T3), despite the absence of a lockdown.

## 4. Discussion

The COVID-19 pandemic has had a major impact on higher education, with university students being subjected to constraints that abruptly changed their lifestyles. Although we have some knowledge of the changes that resulted from the first lockdown, we have limited evidence of how students’ lifestyles have changed since then. The main objective of the present study was therefore to describe the modifications in sedentary behaviors, physical activity, and alcohol consumption among French university students by conducting measures at four different timepoints (two lockdowns and the intervening period).

First, the results showed a decrease in alcohol consumption during both lockdowns, consistent with previous studies conducted among students [15,16,17]. However, our results are in opposition to the findings from previous disasters (e.g., natural disasters and attacks), which reported an increase in alcohol consumption [31]. These differences may be due to the fact that the COVID-19 pandemic is an exceptional situation, never encountered before, which is a complex multi-faceted experience affecting billions of people worldwide. In addition, the effects of the pandemic on consumption vary by age [12,32]. More precisely, individuals who declared low alcohol consumption during lockdown were significantly younger (a mean of approximately 26 years) than the rest (mean of above 30 years) [12]. According to these authors, this may be because pubs, clubs, and halls of residence were closed and classes suspended during lockdown, thus lessening the opportunity of meetings between young adults as they had to stay at home. Indeed, the restrictive context of the lockdowns prevented students from meeting to drink together—the primary mode of consumption among youth adults [33]. Students probably had fewer opportunities to consume alcohol during the lockdowns, especially those living at home with their parents, who may have found that their drinking habits were not endorsed by their family and adjusted their alcohol consumption accordingly [34].

Second, the results for physical activity revealed different trajectories depending on the intensity of the activity. To date, most studies of students’ physical activity have asked them to compare their physical activity levels with questionnaires administered retrospectively (for pre-pandemic) and prospectively (during the pandemic) [20,22]. Our study is one of the few that measured the evolution of students’ physical activity at different time points during the COVID-19 pandemic, as well as specifying this evolution according to the intensity of the activities. More specifically, our participants had very high levels of light activity (e.g., walking slowly or household activities) during the first lockdown, which was also observed in Spanish students, but for all types of physical activities [25]. This can be explained by the fact that lockdown meant people spent more time inside their homes and performed these types of activities [35]. However, the level of light physical activity fell dramatically after the first lockdown and remained low during the second lockdown. Thus, this increase was not sustained over time, indicating the lack of a lasting change in students’ habits. This can be attributed to a loss of motivation or even resignation due to the length of the pandemic.

Third, our results showed that sedentary behavior levels were higher during the lockdowns and the restart of the academic year (i.e., partly composed of distance learning courses) than during the summer break. Distance learning has drastically increased students’ sedentary behaviors [20,36], both because they remain seated throughout teaching hours and because there is no longer any travel to and from university between classrooms or during breaks. Given the widely demonstrated deleterious effects of sedentary behaviors on health [37], it is essential that universities initiate actions aimed at combating them among students, especially as the latter were already found to have sedentary behaviors before the pandemic [6,7]. This is reflected in our results, which showed an increase in sedentary behaviors when the academic year resumed (T3) compared to the summer period (T2). In other words, the pandemic and distance learning have simply highlighted an issue that was already present. In 2015, the Okanagan Charter [38] identified universities as an important setting for health promotion, and it is crucial to follow and promote this charter in the current situation. For example, face-to-face or online health promotion and disease prevention campaigns should be used to raise students’ awareness of the importance of physical activity [39].

The present study has several limitations, meaning that some results should be interpreted with caution. First, despite a large sample at the first timepoint, only 91 participants responded to the four measurement times over the seven months and could be included in this study. In our study, the large loss of participants between T1 and T2 may in part be explained by the end of the academic year, a less frequent checking of their emails, or a voluntary detachment from the academic environment. Second, because our second assessment was conducted at the beginning of the summer vacation, it was difficult to dissociate the effects of coming out of lockdown and being on vacation. Third, our measure of alcohol consumption before the pandemic relied on participants’ memory, which may have biased some responses. Furthermore, our study relied on a subjective measure of physical activity, and it would be interesting to supplement this with an objective assessment (e.g., using accelerometers). Further research is needed to confirm these trajectories and to explore the duration of these changes in students’ lifestyles over time.

## 5. Conclusions

To the best of the authors’ knowledge, this is the first study conducted on university students during four different periods of the COVID-19 pandemic and over seven months. This research highlighted changes in students’ lifestyles, some of which may have a negative impact on their long-term health. These results indicate that university students, who are known to constitute a vulnerable population, have become even more vulnerable during the pandemic. This leads us to worry about the long-term effects of the pandemic on students’ sedentary behaviors and physical activity. In the future, it will be essential for universities to promote active breaks and provide more opportunities for students to engage in regular physical activity.

## Figures and Tables

**Table 1:** Characteristics of the survey respondents.

Characteristics	Total (N = 91)
	Number (%)
**Sex**	
Female	67 (73.62)
Male	18 (19.78)
Other	6 (6.59)
**University**	
Nîmes	46 (50.54)
Lorraine	26 (28.57)
Strasbourg	11 (12.08)
Other	8 (8.79)
**Education level**	
Undergraduate	79 (86.81)
First year	30 (32.97)
Second year	27 (29.67)
Third year	22 (24.18)
Master’s	10 (10.99)
Fourth year	9 (9.89)
Fifth year	1 (1.10)
PhD	2 (2.20)
**Place of residence**	
Parental home	53 (58.24)
Own accommodation	38 (41.76)

**Table 2:** Repeated measures ANOVA effects.

Variables	df	F	*p*	η²
Alcohol	4	8.83	<0.001	0.11
Sedentary behaviors	3	4.59	0.004	0.05
Physical activity	3	14.7	<0.001	0.14
Light	3	59.35	<0.001	0.40
Moderate	3	4.49	0.005	0.07
Vigorous	3	2.56	0.06	0.12

**Table 3:** Post-hoc comparison.

		Alcohol		Sedentary Behaviors		Physical Activity		Light		Moderate		Vigorous	
		MD	*t*	MD	*t*	MD	*t*	MD	*t*	MD	*t*	MD	*t*
Before pandemic	T1	0.81	3.86 ***										
	T2	0.14	0.66										
	T3	0.29	1.38										
	T4	1.01	4.85 ***										
T1	T2	−0.67	−3.21 **	0.90	2.71 *	4.46	5.06 ***	4.52	11.41 ***	−1.13	−1.70	−1.37	−1.39
	T3	−0.52	−2.49	−0.02	−0.07	2.47	2.80 *	4.21	10.63 ***	−1.57	−2.37	−0.87	−0.88
	T4	0.21	0.98	−0.23	−0.68	5.39	6.12 ***	4.19	10.57 ***	0.59	0.89	1.18	1.19
T2	T3	0.15	0.72	−0.92	−2.78 *	−1.99	−2.26 *	−0.31	−0.78	−0.44	−0.66	0.50	0.51
	T4	0.88	4.19 ***	−1.13	−3.39 **	0.94	1.06	−0.33	−0.84	1.71	2.59	2.55	2.58
T3	T4	0.73	3.47 **	−0.20	−0.61	2.93	3.32 **	−0.02	−0.06	2.15	3.25 **	2.05	2.08

Note: MD, mean difference; * *p* < 0.05, ** *p* < 0.01, and *** *p* < 0.001.

## Supplementary Materials

The following are available online at https://www.mdpi.com/article/10.3390/ijerph18178998/s1, Table S1: Characteristics of survey respondents at the four timepoints.
