# Supplementary material for: University Students’ Lifestyle Behaviors during the COVID-19 Pandemic: A Four-Wave Longitudinal Survey
Source: Int J Environ Res Public Health. 2021 Aug 26;18(17):8998. doi: 10.3390/ijerph18178998 (PMC8430950; doi:10.3390/ijerph18178998)
Supplement: Supplementary file 1 [file ijerph-18-08998-s001.zip › ijerph-1323119-supplementary.pdf]

International Journal of
Environmental Research and Public Health

MDPI

Table S1. Characteristics of survey respondents at the four timepoints.

Characteristics	T1 (N = 1294)	T2 (N = 373)	T3 (N = 284)	T4 (N = 160)
	Number (%)	Number (%)	Number (%)	Number (%)
**Sex**				
Female	1006 (77.7)	310 (83.1)	232 (81.7)	124 (77.5)
Male	268 (20.7)	54 (14.5)	44 (15.5)	29 (18.1)
Other	20 (1.6)	9 (2.4)	8 (2.8)	7 (4.4)
**University**				
Nîmes	558 (43.12)	179 (47.99)	142 (50)	78 (48.75)
Lorraine	370 (28.59)	82 (22.25)	70 (24.65)	47 (29.38)
Strasbourg	212 (16.38)	64 (17.16)	39 (13.73)	23 (14.38)
UCO Angers	86 (6.65)	20 (5.36)	12 (4.23)	5 (3.13)
Others	68 (5.26)	29 (7.77)	21 (7.39)	7 (4.38)
**Education level**				
Undergraduate				
First year	486 (37.56)	100 (26.81)	96 (33.80)	57 (35.63)
Second year	314 (24.27)	106 (28.42)	72 (25.35)	47 (29.38)
Third year	323 (24.96)	98 (26.27)	69 (24.30)	35 (21.88)
Master’s				
Fourth year	82 (6.34)	34 (9.12)	27 (24.30)	15 (9.38)
Fifth year	74 (5.72)	26 (6.97)	13 (4.58)	3 (1.88)
PhD	11 (.85)	8 (2.14)	6 (2.11)	3 (1.88)
Undefined	4 (.31)	2 (.54)	1 (.35)	0
**Place of residence**				
Parental home	911 (70.40)	215 (57.64)	78 (27.46)	73 (45.63)
Own accommodation	383 (29.60)	158 (42.36)	206 (72.54)	87 (54.37)

Note. Concerning the level of education, numbers are based on participants’ responses at T1.
